# Supplementary material for: Controlled Thermal Release of L-Menthol with Cellulose-Acetate-Fiber-Shelled Metal-Organic Framework
Source: Molecules. 2022 Sep 15;27(18):6013. doi: 10.3390/molecules27186013 (PMC9502463; doi:10.3390/molecules27186013)
Supplement: Supplementary file 1 [file molecules-27-06013-s001.zip › molecules-1904146-supplementary.pdf]

Supplementary for
Controlled Thermal Release of L-Menthol with Cellulose Acetate Fiber Shelled Metal-Organic Framework

Xinjiao Cui[1,#], Donghao Ye[2,#], Jiankun Wei1, Xiaodi Du[1,*] Pengzhao Wang[3,*] and Junsheng Li[1,*]

1 School of Chemistry, Chemical Engineering and Life Sciences, Wuhan University of Technology, Wuhan 430070, P. R. China
2 Wuhan Marine Electric Propulsion Research Institute, Wuhan 430064, P. R. China
3 College of Chemical Engineering, Fuzhou University, Fuzhou 350108, P. R. China
* duxiaodi@msn.com (X. Du), wpz@fzu.edu.cn (P. Wang), li_j@whut.edu.cn, (J. Li).

Table S1. Shifts in corresponding IR peaks of MIL-101 (Cr) and LM@ MIL-101 (Cr)

Assignment	MIL-101 (Cr)	LM@MIL-101 (Cr)
OH- stretching vibration	3450 cm^{-1}	3380 cm^{-1}
-C-H, -CH_3, and -CH_2 asymmetric stretching vibrations	/	2960 cm^{-1}
-C-H, -CH_2 asymmetric stretching	/	2930 cm^{-1}
-C-H symmetric stretching vibrations	/	2870 cm^{-1}
C=C stretching vibration	1630 cm^{-1}	1630 cm^{-1}
C=C stretching vibration	1500 cm^{-1}	1500 cm^{-1}
O-C-O symmetric stretching vibration	1380 cm^{-1}	1400 cm^{-1}
C-H stretching vibration	1050 cm^{-1}	1050 cm^{-1}
C-H stretching vibration	1020 cm^{-1}	1020 cm^{-1}
C-H stretching vibration	750 cm^{-1}	747 cm^{-1}
Cr-O stretching vibration	550 cm^{-1}	592 cm^{-1}

Table S2. BET date analysis of MIL-101 (Cr) and LM@ MIL-101 (Cr)

Samples	BET Area	Micropore Area	Micropore volume
MIL-101 (Cr)	2636.8 $m^2.g^{-1}$	406.8167 $m^2.g^{-1}$	1.06 $cm^3.g^{-1}$
LM@MIL-101 (Cr)	1344.8 $m^2.g^{-1}$	386.8031 $m^2.g^{-1}$	0.56 $cm^3.g^{-1}$

Table S3. Pseudo-first order and pseudo-second order kinetic fitting parameters

Modle	q_e	k	R^2
Pesudo-first order	432.1 mg g^{-1}	4.46 min^{-1}	0.996
Pesudo-second order	448.8 mg g^{-1}	0.02 g $(mg.min)^{-1}$	0.980

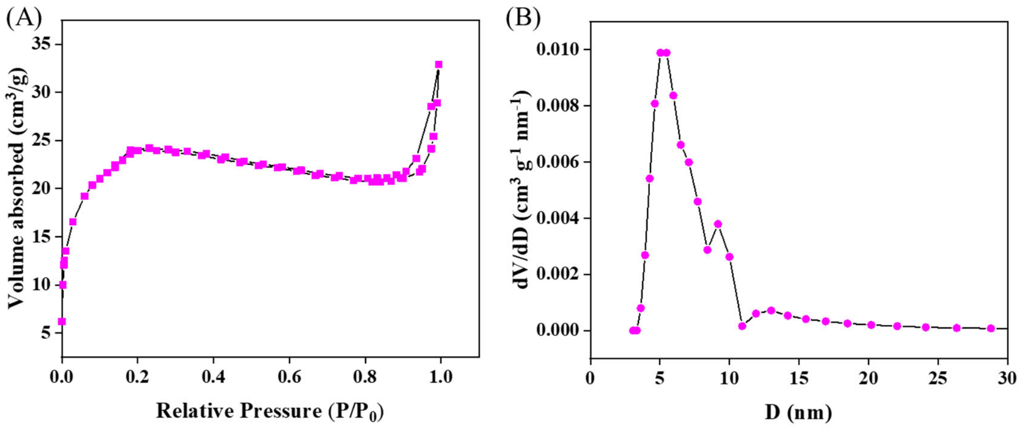

Figure S1. (A) N_2 adsorption/desorption isotherms; (B) Pore size distribution of LM@MIL-101 (Cr)-$CAF_{0.9}$.

Table S4. Heat release of LM in different systems

Samples	The amount of LM released (μg)							
	32s	64s	96s	128s	160s	192s	224s	256s
**LM**	14.41	15.29	16.68	14.81	13.46	12.81	12.60	11.79
**LM-$CAF_{0.9}$**	9.11	11.73	8.69	8.10	6.89	6.38	5.98	5.59
**LM@MIL-101 (Cr)-$CAF_{0.7}$**	6.69	8.40	8.14	7.30	6.79	6.82	5.98	5.64
**LM@MIL-101 (Cr)-$CAF_{0.9}$**	10.29	11.56	10.77	10.95	10.74	9.67	9.12	8.59
**LM@MIL-101 (Cr)-$CAF_{1.1}$**	8.06	7.71	7.23	7.96	7.11	7.76	7.31	7.23
**LM@MIL-101 (Cr)-$CAF_{1.3}$**	6.58	8.18	7.72	8.08	7.71	7.62	7.20	7.22
